# DrugEx: Deep Learning Models and Tools for Exploration of Drug-Like Chemical Space

**DOI:** 10.1021/acs.jcim.3c00434

**Published:** 2023-06-05

**Authors:** Martin Šícho, Sohvi Luukkonen, Helle W. van den Maagdenberg, Linde Schoenmaker, Olivier J. M. Béquignon, Gerard J. P. van Westen

**Affiliations:** † Leiden Academic Centre for Drug Research, Leiden University, 55 Einsteinweg, 2333 CC, Leiden, The Netherlands; ‡ CZ-OPENSCREEN: National Infrastructure for Chemical Biology, Department of Informatics and Chemistry, Faculty of Chemical Technology, University of Chemistry and Technology Prague, Technická 5, 166 28, Prague, Czech Republic

## Abstract

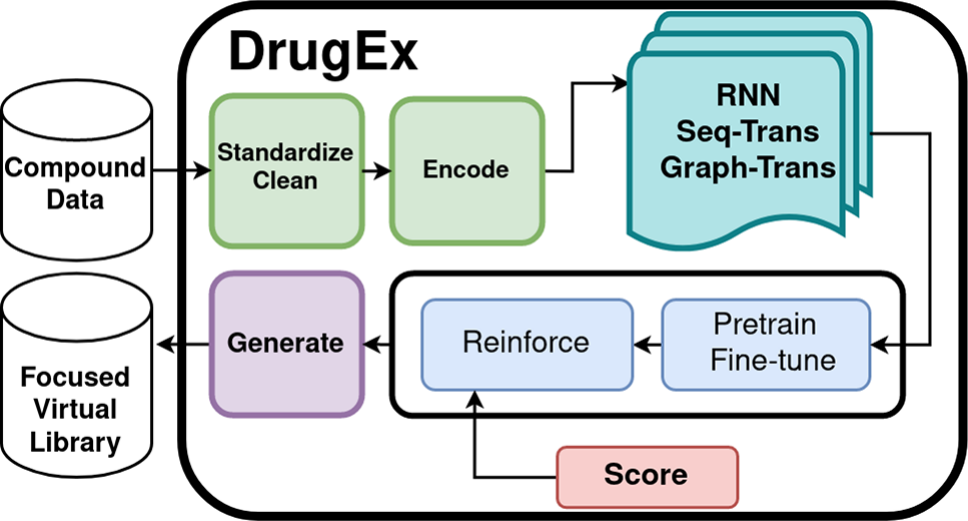

The discovery of novel molecules with desirable properties is a classic challenge in medicinal chemistry. With the recent advancements of machine learning, there has been a surge of *de novo* drug design tools. However, few resources exist that are user-friendly as well as easily customizable. In this application note, we present the new versatile open-source software package DrugEx for multiobjective reinforcement learning. This package contains the consolidated and redesigned scripts from the prior DrugEx papers including multiple generator architectures, a variety of scoring tools, and multiobjective optimization methods. It has a flexible application programming interface and can readily be used via the command line interface or the graphical user interface GenUI. The DrugEx package is publicly available at https://github.com/CDDLeiden/DrugEx.

## 1 Introduction

Drug discovery is a tedious and resource-intensive process that can take decades and on average costs millions of dollars.^1^ Computer-aided drug design facilitates this process by selecting promising compounds over ones with a poor prognosis. Using *de novo* drug design (DNDD), novel hit, lead, and future drug candidates can be found by exploring the vastness of the drug-like chemical space (∼10^63^ molecules).^2^

Rapid technological improvements over the last decades have led to the rising popularity of advanced machine learning methods.^3−5^ These developments have also greatly influenced the field of DNDD with state-of-the-art methods including population-based metaheuristics, recurrent neural networks (RNNs), generative adversarial networks, variational autoencoders, and transformers.^6−8^ Moreover, concepts such as transfer, conditional, and reinforcement learning (RL) are often applied to generate molecules with desired properties.

Typical objectives guiding the drug discovery process are maximization of predicted efficiencies, synthetic accessibility or drug-likeness of the compounds, and minimizing off-target effects and toxicity. Even without optimization toward favorable physicochemical and pharmacokinetic properties, DNDD is inherently a multiobjective optimization (MOO) problem.^7,9^

In this application note, we present the new open-source software library DrugEx, a tool for *de novo* design of small molecules with deep learning generative models in a multiobjective RL framework. This comprehensive tool represents the consolidation of the original work of Liu et al.’s multiple scripts based DrugEx releases. The first version of DrugEx^10^ consisted of an RNN single-task agent of gated recurrent units (GRU) which were updated to long short-term memory (LSTM) units in the second version,^11^ also introducing MOO-based RL and an updated exploitation-exploration strategy. In its third version,^12^ generators based on a variant of the transformer^13,14^ and a novel graph-based encoding allowing for the sampling of molecules with specific substructures were introduced. These developments were built on the work of Olivecrona et al.^15^ for the use of reinforcement learning and those of Arús-Pous et al.^16^ and Yang et al.’s *SynthaLinker*(17) for the recurrent neural network and transformer architectures, respectively.

In Section 2.1, we describe the currently available generator algorithms, the different training modes, and data preprocessing steps and amend the recently introduced graph encoding of molecules from ref (12). In Section 2.2, we present the three steps to score compounds for the RL, the computation and scaling of scores per objective, the multiobjective optimization, and detail some predefined options. Furthermore, to facilitate usage, this work is supplemented with a rich Python application programming interface (API), a command line interface (CLI), and a graphical user interface (GUI) that are described in Section 3. Finally, pretrained models are made publicly available to ease the *de novo* design of molecules. This application note gives an overview of all these capabilities that have been consolidated in the DrugEx package. Thereby allowing users to more easily apply *de novo* drug design techniques and customize them in the way they see fit.

## 2 Application Overview

### 2.1 Molecular Generator

#### 2.1.1 Algorithms

The original DrugEx articles describe six different generator architectures.^10−12^ The current DrugEx package includes four of these models: two SMILES-based recurrent neural networks using GRU or LSTM units and sequence- and graph-based transformers using fragments as input. The fragment-based LSTM models with and without attention from ref (12) have been discontinued as they were outperformed by the other models. The available models are briefly introduced below, and the detailed model architectures are described in Section S1.

#### Recurrent Neural Networks

RNNs are used to create molecules without the use of input fragments. These molecules are generated in the form of tokenized SMILES sequences. The RNNs are built from LSTM units^18^ or GRUs.^19^ The RNN model consists of the following layers: an embedding layer, three recurrent layers, a linear layer, and a softmax activation layer. These building blocks are trained to predict the most likely next output token. Compared to the transformer models, this generator does not require inputs, is quick to train, and still has a relatively low error rate.

#### Transformers

In addition to the token-based RNNs, DrugEx includes two fragment-based models that are variants of the transformer model using either graphs or sequences as molecular representations. For the fragment-based modeling, molecules are constructed from building blocks (detailed in Section 2.1.2). These fragments are combined to create fragmented scaffolds, which form the input for the model, and are grown into novel molecules.

#### Sequence-Based Transformer

The sequence-based transformer model is a decoder-only transformer that applies a multiheaded attention architecture and position-wise feed-forward layers followed by a linear layer and an activation function to predict the most likely output token.^13,14^ In contrast to the RNN, the transformer models allow for user-defined inputs in the form of fragments. Furthermore, contrary to the graph-based transformer, the sequence-based transformer allows for the direct incorporation of stereochemistry defined by the molecular notation used.

#### Graph-Based Transformer

The graph-based transformer variant deals with the positional encodings differently from the more classical sequence-based transformer encodings.^13^ As with a graph representation, the atom index cannot directly be used, and the encoding is a combination of the atom index (current position) and the connected atom index (previous position).^12^ For more details on the graph representation of the molecules, see Section 2.1.2. The graph-based model consists of a transformer encoder and a GRU-based decoder. The graph transformer has some considerable advantages compared to the sequence-based transformer as it creates only valid molecules and has a higher incorporation rate of fragments.

#### 2.1.2 Data Preprocessing

The following section describes the default implementation of molecular preparation, i.e., standardization and fragmentation in DrugEx (detailed information in Section S2). Nevertheless, custom steps can easily be implemented with the provided Python API (Section 3.1).

**Figure 1:**
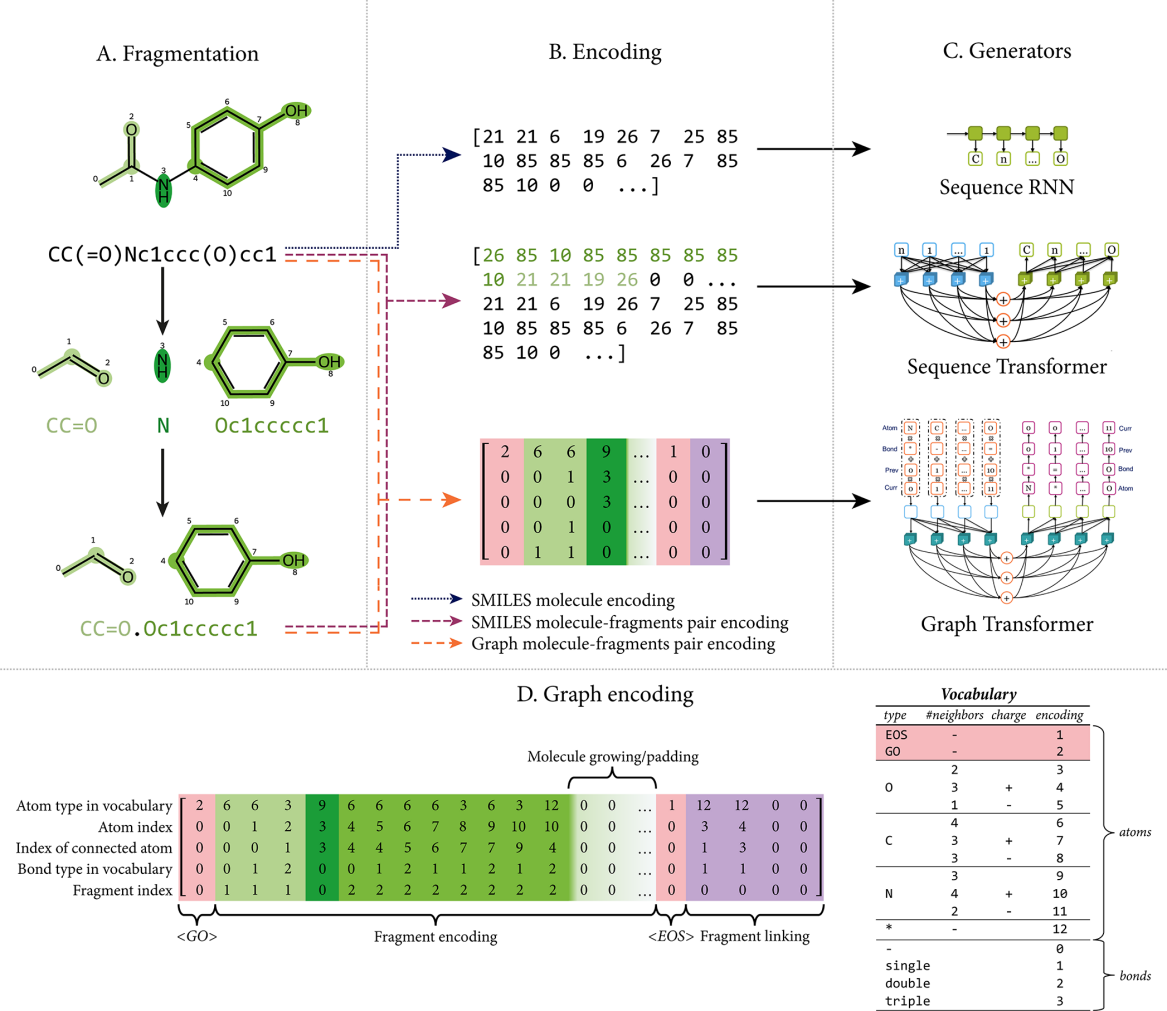
Correspondence of input and encoding types with generator models. Input molecules are fragmented for sequence and graph transformers (A), and then input molecules or molecule-fragment pairs are encoded (B) before being used for training and/or sampling by the three generator architectures available (C). Graph encoding matrix of acetaminophen (D) based on the vocabulary next to it. To be encoded as a graph, the molecule is split into three fragments by the BRICS algorithm along the bonds on both sides of the nitrogen atom. Based on the atom-type and bond-type vocabulary encodings, a graph matrix is constructed. This matrix consists of five rows: (i) the current atom type as encoded by the vocabulary, (ii) the 0-based atom index in the molecule, (iii) the index of one of its neighboring atoms (if starting a fragment the index of the atom itself), (iv) the bond type as encoded by the vocabulary, and (v) the 1-based index of the fragment being encoded, respectively. The matrix consists of four major column blocks, from left to right, the start token block (⟨GO⟩, pink), the columns used for the encoding of fragments (green), the end token block (⟨EOS⟩, pink), and columns indicating the linking between fragments (purple). The dimension of the graph matrix is  with , where *n*_fragments_ is the number of fragments encoded in the molecule and *d* the width of the block encoding fragments. Should *d* be greater than the number of columns required to encode all fragments of a molecule, the remaining columns are filled with zeros, as exemplified by the sub-block used for padding. During sampling, this sub-block is used to grow the molecule. By default, the graph matrix has dimensions 5 × 400 theoretically allowing for the encoding of molecules with molecular weights of up to 10,000 g/mol.

In short, standardization is applied, ensuring that only organic molecules are kept. Then, for the transformer models, fragmentation is performed using the BRICS^20^ or RECAP^21^ algorithms. Combinations of the obtained fragments are made for the model to be pretrained or fine-tuned on hybridizing these fragments to form the original molecules. For the RNN, no fragmentation is performed as the model creates molecules from an empty solution. Encoding of the inputs differs between sequence- and graph-based models. Figure 1 illustrates the encoding types per generator algorithm and gives a detailed description of the graph-based encoding as it amends that described in ref (12). Details on the encoding of SMILES sequences are available in Section S2. Both the fragmentation and encoding processes determine the minimum and maximum sizes of molecules used for training which are between 200 and 1000 Da, with default parameters (Figure S3).

#### 2.1.3 Training

#### Pretraining and Transfer Learning

Before guiding a generator to create compounds with specific properties, it needs to be pretrained to learn the language of drug-like molecules and be able to generate reasonable molecules. This involves training a generator to reproduce compounds from a large set of (fragmented) drug-like molecules. We have shared pretrained RNN-based and transformer-based generators (pretrained on ChEMBL27, ChEMBL31,^22^ and Papyrus v5.5^23^) on Zenodo.^24^ Further details about the pretrained models are given in Sections S1 and S4.

Furthermore, a generator can be directed toward the desired chemical space by fine-tuning a pretrained generator via transfer learning with a set of molecules occupying the desired chemical space.

During training, the loss on a separate test set is assessed at each training epoch to select the best model epoch and allow for early stopping. In brief, for all models, the loss is calculated by taking the average negative log-likelihood (softmax) of the predicted outputs. For the SMILES-based model, it is also possible to use SMILES validity for this purpose.

#### Reinforcement Learning

During reinforcement learning, the “desirability” of generated molecules is quantified by the environment based on various properties and used as a reward (Section 2.2). The generator is optimized using the policy gradient scheme.^25^

In order to control the exploration rate, molecules are generated based on the output of two generators: a generator that is updated based on the reward function and saved as the final generator (the exploitation network or “agent”) and a static generator (the exploration network or “prior”). The fraction of outputs coming from each generator mimics the mutation rate in evolutionary algorithms and is tunable. Currently, this has been tested with the fine-tuned model as the exploitation network and the pretrained model as the exploration network.^10−12^ To improve exploration, the exploitation network can use the outputs from two networks, of which one is constantly updated based on the reward function and the other only updated every 50 epochs by default (as is shown by Figure 3 in the original paper^11^). This is the default for the RNN-based generator. For the transformer-based generators, due to their higher computation costs, this periodically updated generator is not used by default, and we generally advise against using it with them. A reload interval of 50 epochs seems to provide a good balance between the computational cost of reloading and added benefit of more exploration. However, we have yet to explore the effect of this parameter fully. Multiple metrics are computed at each epoch: the ratio of valid, accurate (only for fragment-based models), unique, or desired molecules, and the average arithmetic and geometric mean score per objective. In addition to this, users can define customized metrics in the API. A compound is “desired” if it fulfils all objectives as defined in Section 2.2.2. One of the metrics, by default the desirability ratio, is used to select the best model epoch and to allow for early stopping. For fragment-based sampling, the inputs can either be a specific scaffold or the unique fragment combinations of a given data set.

### 2.2 Molecule Scoring

The scoring of molecules with the environment at each reinforcement learning epoch is done in three stages: (i) obtaining raw scores for each of the selected objectives (Section 2.2.1), (ii) scaling of the raw scores with modifier functions (Section 2.2.2), and (iii) a multiobjective optimization step (Section 2.2.3) to obtain a final reward per molecule.

#### 2.2.1 Objectives

The API gives a large flexibility to the user to use custom scoring methods that take SMILES as inputs and give a numeric score as output. Moreover, DrugEx is coupled with the QSPRpred package to allow the use of a wide range of ML models and offers a range of other predefined objective functions.

#### QSPRpred

To optimize the binding affinity for one or more targets or other molecular properties, DrugEx users can choose to add one or more quantitative-structure activity/property (QSAR/QSPR) models as objectives for the reinforcement learning reward. To this end, DrugEx is compatible with any Python script that receives SMILES as input and produces a score as an output through the API. A separate package, QSPRpred (https://github.com/CDDLeiden/QSPRPred), was developed to simplify the development of QSAR models. The setup of QSPRpred is very similar to DrugEx, using the same structure of the API and CLI. It also comes with tutorials to help users get started. QSPRpred has a selection of scikit-learn^26^ models and a PyTorch^27^ fully connected neural network available through the API, so the user can train a wide variety of QSAR models. Furthermore, due to its modularity, QSPRpred is customizable; users can for example add new model types and molecular descriptors.

#### Predefined Objectives

The DrugEx package offers a set of predefined property calculations that can be used as objectives in the scoring environment. These components are summarized in Section S3 and include functions to compute ligand or lipophilic efficiencies from affinity predictions, a variety of similarity measures to a reference structure, estimations of (retro)synthetic accessibility, and a plethora of physicochemical descriptors.

#### 2.2.2 Modifiers

To ensure the optimization, each objective is coupled with a modifier function that transforms it into a maximization task and scales all raw scores between 0 and 1. Custom modifiers are easily implemented with the API, but DrugEx offers a variety of predefined modifiers for both monotonic (e.g., ClippedScore) and nonmonotonic objectives (e.g., Gaussian). Some of these modifiers do not normalize or do not transform the objectives to maximization tasks in all cases and should be used with caution, especially when using an aggregation-based multiobjective optimization scheme. All modifiers are summarized in Section S3. Each objective–modifier couple is associated with a desirability threshold set between 0 and 1 to determine if a compound fulfills the desirability criteria on that objective or not. A compound is considered desired if for all objectives its modifier scores are above their corresponding thresholds.

#### 2.2.3 Multiobjective Optimization

Since version 2, DrugEx enables multiobjective optimization during RL.^11^ DrugEx offers three different MOO schemes: a parametric aggregation method and two Pareto ranking-based schemes.^7,9^ The aggregation method is the parametric weighted sum (WS) which uses dynamic weights for each objective to especially reward compounds that perform well on the worst-performing objective(s) at each iteration.^11^ Pareto-based schemes do not combine multiple objectives into one but rather search for the best trade-off between them, and the initial ranking of molecules is done based on the ranking of the Pareto frontiers. After assigning each molecule to a front, the compounds in each frontier are ranked based on a distance metric to increase the diversity of solutions.

DrugEx proposes two distance metric formulations: the crowding distance (PRCD)^28^ and the Tanimoto distance (PRTD).^11^ Crowding distance is commonly used in multiobjective optimization tasks and is designed to increase the diversity of solutions with regards to how they fulfill each objective and, thus, reflects the diversity of solutions purely by their position in the objective space. In the context of molecular generation, this can sometimes be at the expense of molecular diversity. Therefore, several subtly different ranking schemes were devised utilizing the Tanimoto distance, which relates to the structural diversity of the molecules in the front. Detailed descriptions of the three schemes are given in Section S3.

The choice of an appropriate optimization scheme depends on the task at hand and usually requires some level of experimentation. The WS is more stable for many-objective optimization than the Pareto-based methods as the number of Pareto-equivalent solutions increases with the number of objectives. On the other hand, the Pareto-based schemes enforce diversity in the ranking which is not the case for the WS. The computational complexity of the WS is lower than the Pareto’s and is much faster. However, the ranking computation time (below 1 s for 10,000 molecules with three objectives with both approaches) is negligible compared to the other steps of the reinforcement learning training (generation, decoding, scoring).

## 3 Implementation

Aside from the addition of several new features for the generation and scoring of molecular structures, a significant part of the development was dedicated to creating a flexible and scalable software architecture. We have extensively revised the original Python source code of all published DrugEx models^10−12^ and transformed it into a self-contained open-source Python package with a clear structure and API. In addition, a simple CLI was also implemented that allows quick invocation of the main DrugEx functions and improves the management of inputs and outputs. The package supports many monitoring utilities that log training progress and result in easy-to-read machine-readable formats such as TSV (Tab Separated Values) and JSON (JavaScript Object Notation) files. When using the CLI, these files are backed up after each (even unsuccessful) run so older results and settings are not lost and can be retrieved at any time. These and other modifications should empower users to quickly explore different scenarios when building their generative models and also ensure reproducible results by keeping track of the set parameters. Both the CLI and Python API are documented, and we also created easy-to-follow Jupyter notebooks tutorials to help users get started. Finally, we have performed significant optimizations in multiple parts of the workflow by utilizing multiprocessing where possible.

### 3.1 Python Package

**Figure 2:**
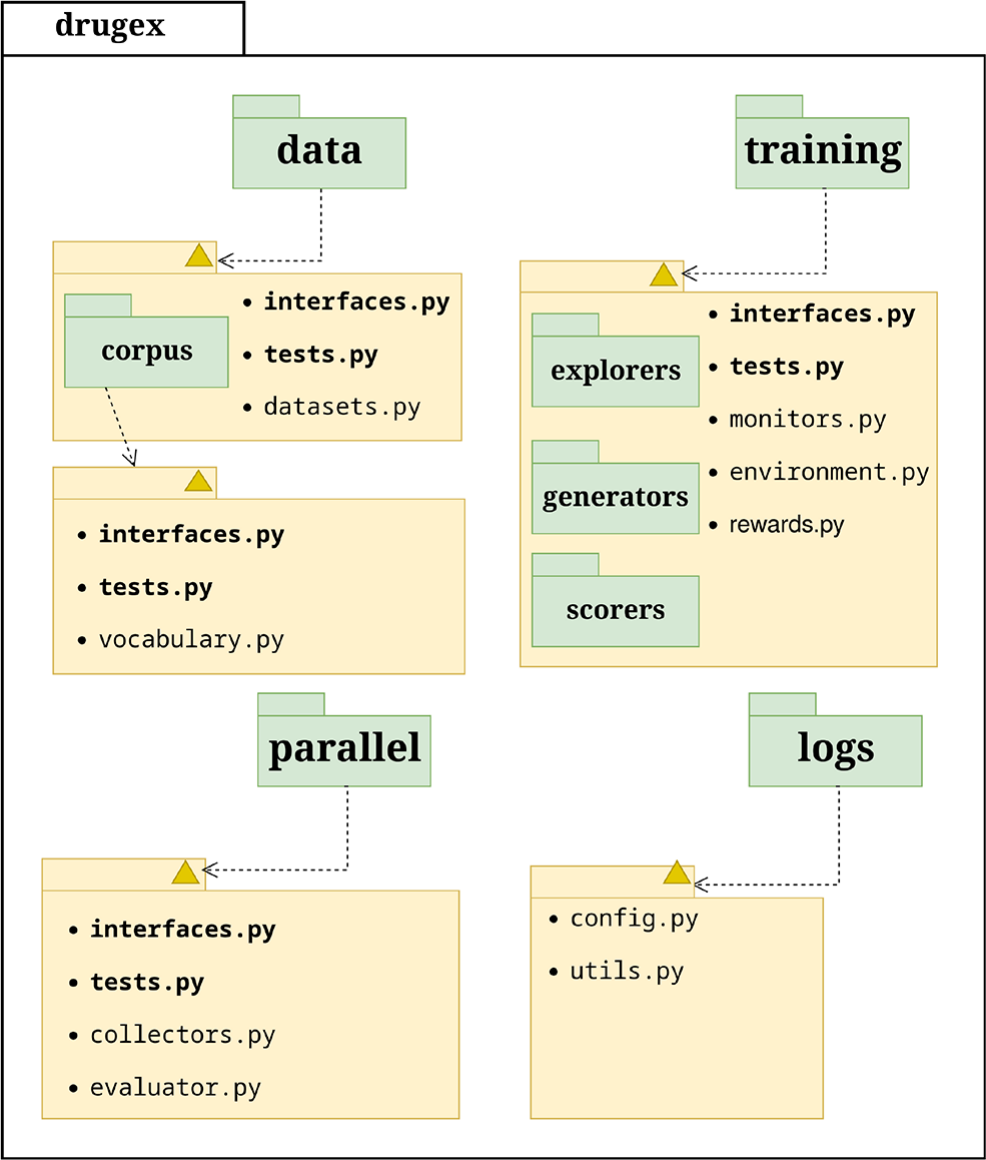
A simplified diagram of the open-source Python package architecture. The two main subpackages of the drugex package are data and training. The data package handles the preparation of data sets that can then be used to instantiate and train models in training. Aside from the model classes themselves (generators), the training package also contains data structures needed to train models with reinforcement learning (explorers) using a scoring environment based on scoring functions (scorers).

The software package is divided into intuitively organized subpackages and modules, each handling either preprocessing of the data, model training, or generation of new molecules by loading the model for sampling (Figure 2). The code is organized with modularity, extensibility, and testability in mind. Each larger subpackage contains a clear definition of its interfaces in the interfaces.py module and also unit tests in tests.py. Interface definitions are most of the time facilitated through abstract classes for which each subpackage provides default implementations that can be automatically tested with the provided unit tests. These classes form the core of the DrugEx Python API that users can exploit to make modifications to their workflows and/or interact with the DrugEx models programmatically.

#### Application Programming Interface

The Python API exposes many functions from preprocessing, model training, and sampling of molecular structures. Users can mix and match the necessary objects or create custom classes to accomplish their goals. For example, it is possible to apply customized fragmentation strategies by implementing the fragmentation API or use a modified training monitor class to change progress and result tracking during model training. All components of the project follow the principles of object-oriented programming to make implementations of such extensions possible without changing the code of the package itself. Examples of such usage are also available in the tutorials (see Tutorials).

#### Command Line Interface

If no customization is required, the package also offers a CLI for quick setup of experiments with default implementations of the most common tasks. The package contains three main executable scripts: (1) dataset.py, (2) train.py, and (3) generate.py. These scripts are usually executed in order to preprocess input data, train new models, and generate a virtual library of compounds. These scripts are installed with the package drugex. Each script also automatically logs standard input and output, tracks the history of executed commands, and stores generated data outputs so that they can be retrieved later which adds to the reproducibility of experiments.

#### Documentation

Both API and CLI usage is documented, and we have tried to provide a sufficient description of each interface, class, and function. This Sphinx-generated documentation is available at https://cddleiden.github.io/DrugEx/docs/ and is updated with each new DrugEx release.

#### Tutorials

Aside from source code documentation, the DrugEx web page also provides descriptions of command line arguments and usage examples for the CLI. In addition, we also compiled a collection of Jupyter notebooks that provides a comprehensive introduction to the Python API. The tutorials feature more advanced concepts and are a good starting point for any users who require more customization or any future contributors to familiarize themselves with the code.

#### Hardware Requirements

DrugEx offers models of different complexities, and thus, the hardware requirements vary with each model. To be able to train and use all models in the package, the user needs at least one GPU compatible with CUDA 9.2 and at least 8 GB of video memory to save the model and sufficiently large training batches. However, the basic sequential RNN model should be possible to fine-tune and optimize with reinforcement learning even on a less optimal configuration. For the two transformers, we recommend using multiple GPUs to increase throughput by parallelization, which is automatically handled by the package. The GPUs used herein are detailed in the caption of Table S3 in Section S4

#### Contributions to the Open-Source Code

The project embraces the open-source philosophy and welcomes all contributions, questions, or feature requests. The project’s GitHub page contains all the necessary information for potential contributors. In summary, users are advised to first initiate a community discussion on the project’s public issue tracker on GitHub. Subsequently, when an agreement is reached, the contribution can be made through a pull request.

### 3.2 Graphical User Interface

**Figure 3:**
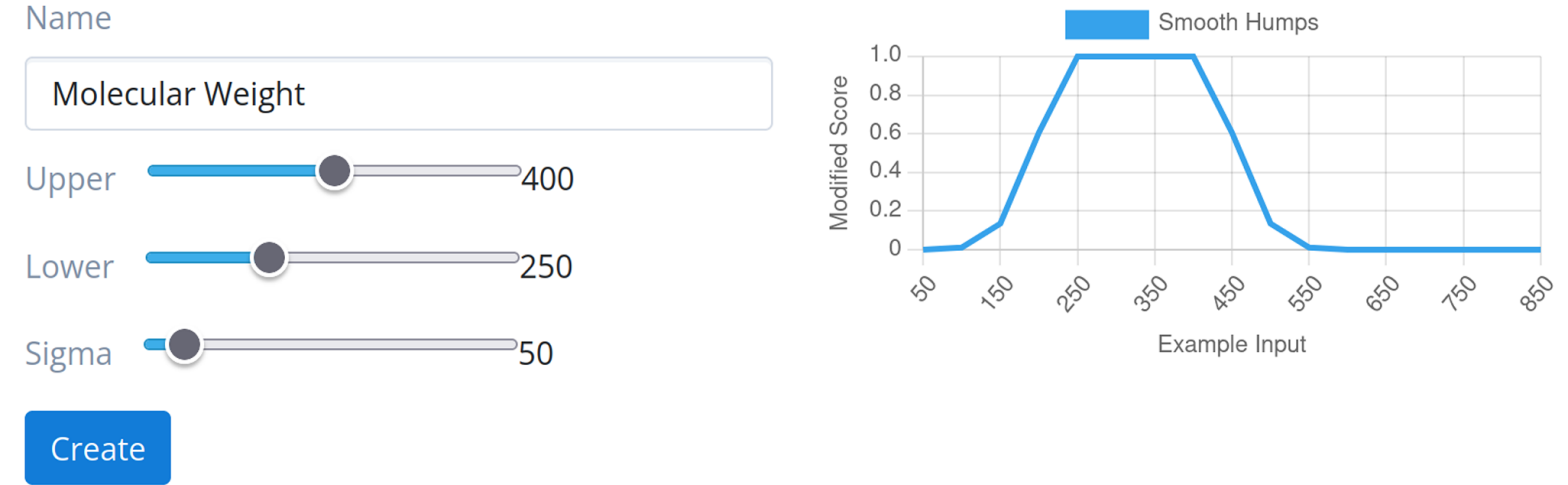
An impression of GenUI interactive interface for visualizing and creating desirability modifiers. The shown view is displayed after the user selects a modifier function and a range of values for its visualization. The modifier function parameters are then simply adjusted with sliders or inputting parameter values directly into input fields (not shown). In the shown example, a Smooth Hump function is used to give maximum score to structures with molecular weight between 250 and 400 Da.

We also added support for the new DrugEx features to our GenUI platform,^29^ which provides a graphical user interface for molecular generators. GenUI is an open-source web-based application built with the Django web framework^30^ and the RDKit cheminformatics toolkit.^31^ GenUI provides features for easy integration of cheminformatics tasks commonly used in *de novo* generation of molecules (i.e., management of a compound database, QSAR modeling, and chemical space visualization). As of now, most of the features available through the DrugEx Python package are also exposed in this GUI to allow quick creation and management of generative workflows from the import of the training data to interactive visual analysis of the generated and real chemical space. One notable feature of the new GUI is the interactive creation of scoring environments, which makes the setup of desirability modifier functions for the multiobjective optimization more intuitive (Figure 3). GenUI is available via prebuilt Docker images and, thus, can be readily deployed in-house or online in virtual environments. Its scalable client-worker architecture also makes it easy to add or remove computation nodes as needed even while the application is running.

## 4 Conclusion

In this paper, we have described the DrugEx open-source software package that facilitates the training of a diverse set of generative models for *de novo* design of small molecules. The package is based on the original Python scripts previously introduced by Liu et al. that were used to develop and validate these models.^10−12^ It includes the following new features: early stopping in all training modes, additional predefined scoring functions, and improved QSPR modeling (hyperparameter optimization, new input features, etc.) with the separate QSPRpred package. The performance has also been enhanced by utilizing parallel processing where possible in both the DrugEx and QSPRpred packages. Furthermore, the current implementation features major revisions of the original API source code of which most notable are the addition of a CLI and Python API. A GUI is also provided via the GenUI web application.

We envisage that the new DrugEx software package and its GenUI integration should be suitable for a diverse set of users. On one side, the package provides a quick and easy way to set up experiments and build models via the CLI and GUI, but on the other side, it also enables more advanced alterations to the workflow through the new Python API. The documentation was also significantly improved, and we now provide easy-to-follow tutorials for new users. Finally, all software presented in this work is provided as open-source software and accessible at https://github.com/CDDLeiden/DrugEx.

We regard the publication of this package as an important step in the development of DrugEx that will be the basis for many research projects and innovations yet to come. In fact, we believe that groundbreaking approaches are only possible when developers of generative models for chemistry undertake such open-source software development initiatives to facilitate prospective validation and testing of their new methods and most importantly their application. Additionally, providing rich documentation and tutorials helps to enhance the models’ usability and integration potential, allowing for faster adoption and feedback leading to the development of better AI-powered models and tools.

In future developments of the DrugEx package, we will not only focus on the integration of novel objectives from the drug discovery toolbox (i.e., molecular docking or retrosynthesis prediction), but also on increasing the range of possible inputs to alternative linear representations of compounds (i.e., SELFIES^32^) in sequence-based models or adding support for encoding stereochemistry. Moreover, we would like to focus on the development of user-centric features such as providing an even better learning platform for teaching the underlying concepts of AI-based molecular generation and improving the GenUI integration. The potential of artificial intelligence in drug discovery is tremendous, but integrating these novel tools in current workflows still remains a challenge. We hope that our software package will help to overcome at least some of those challenges.

## Data Availability

The DrugEx software package is accessible at https://github.com/CDDLeiden/DrugEx. Pretrained models are available on Zenodo.^24^ Timings of pretrained models are on Zenodo as well.^33^

## Supporting Information Available

S1: Generator architecture specification. S2: Data preprocessing. S3: Environment specifications. S4: Sampling statistics. (PDF)
