# Supplementary material for: DrugEx: Deep Learning Models and Tools for Exploration of Drug-Like Chemical Space
Source: J Chem Inf Model. 2023 Jun 5;63(12):3629–36. doi: 10.1021/acs.jcim.3c00434 (PMC10306259; doi:10.1021/acs.jcim.3c00434)
Supplement: Supplementary file 1 — ci3c00434_si_001.pdf [file ci3c00434_si_001.pdf]

Supplementary Information - DrugEx: Deep Learning Models and Tools for Exploration of Drug-like Chemical Space

Martin Šícho[a],†,‡ Sohvi Luukkonen[a],† Helle W. van den Maagdenberg[a],† Linde Schoenmaker[a],† Olivier J. M. Béquignon[a],† and Gerard J. P. van Westen*,†

†*Leiden Academic Centre for Drug Research, Leiden University, 55 Einsteinweg, 2333 CC Leiden, The Netherlands*

‡*CZ-OPENSCREEN: National Infrastructure for Chemical Biology, Department of Informatics and Chemistry, Faculty of Chemical Technology, University of Chemistry and Technology Prague, Technická 5, 166 28, Prague, Czech Republic*

E-mail: gerard@lacdr.leidenuniv.nl

[a]These authors have contributed equally to this work.

S1 Generator architecture specifications

RNN with GRU or LSTM units The GRU- or LSTM-based RNN model have the following architecture (Figure S1A). The input dimension of the embedding layer was set to the number of unique tokens in the default vocabulary. The dimension of the embedding layer output was set to 128. For the recurrent layers, 3 stacked GRUS or LSTMs were used with randomly initialised hidden states with a size of 512. LSTM cell states were randomly initialised with a size of 512. This was followed by a linear layer and a softmax activation function. A negative log-likelihood function was used as the loss function which was optimised using the Adam optimiser.

For the available pretrained models the default learning rate was set to 10^{-3}, the batch size to 1,024, and the generator was trained for a maximum of 1,000 epochs, with early stopping after 50 epochs without improvement. All model parameters can be found in the JSON files accompanying the pretrained models.

Sequence-based transformer The sequence-based decoder-only transformer is implemented similarly to the transformer described by Radford et al.[1] (Figure S1B). Sequence tokens are embedded by an embedding layer with an output dimension of 512. To this output, we add the positional encodings, calculated using sinusoidal functions.[2] The transformer has 12 blocks consisting of multi-head attention (with 12 heads), dropout (0.1) and normalisation, followed by position-wise feed-forward (inner dimension 1,024), dropout (0.1) and normalisation. To facilitate connections in the encoder all sub-layers produce outputs of dimension 512. To predict the most likely output tokens a linear layer with input dimension 512 and an output dimension equal to the number of tokens is used, followed by a log softmax function. During training a negative log-likelihood function was used as the loss function which was optimised using the Adam optimiser.

For the available pretrained models the learning rate was defined as

$$\epsilon_k = \epsilon_0 \times d_m^{-0.5} \times min(k^{-0.5}, kw^{-1.5}) \tag{S1}$$

where e_k is the learning rate for step k, e_0 times $d_m^{-0.5}$ is the initial learning rate and w is the number of warm-up steps. The pretrained models are trained with an e_0 of 0.5, and 4,000 warm-up steps, with a batch size of 128 for a total of 20 epochs.

Graph-based transformer The graph-based transformer encoder uses different embeddings compared to the sequence-based transformer (Figure S1C). For the 'word' embeddings the atom type information and bond type information are combined together into the index of the input word (I) (with a unique input for each possible atom and bond type combination, see[2]). The embedding has an output dimension of 512. For the positional encodings both the atom index (current position) and the connected atom index (previous position) are embedded, resulting in a 512 by 100 (maximum length) matrix. This was then combined with the 'word' embeddings. The encoder has 12 blocks consisting of multi-head attention (with 8 heads), dropout (0.1) and normalization, followed by position-wise feed-forward (inner dimension 1,024), dropout (0.1) and normalization. The atom decoder consists of a linear layer and a softmax activation function that takes the hidden vector from the encoder to predict the atom embeddings. The other decoder consists of a GRU cell with input and hidden state dimensions set to 512, which takes specific elements from the graph output (shifted right) as input and the hidden vector from the encoder (or from subsequent steps) as hidden state. The probability of a bond type, previous and current position is decoded sequentially, by giving the embedded atom, 'word' embedding (I) and embedded connected atoms, respectively and applying a linear layer and a log softmax activation function. To facilitate connections all sub-layers produce outputs with dimension 512. For training a negative log-likelihood function was used as the loss function which was optimized using the Adam optimizer. The learning rate was defined using Equation S1.

For the available pretrained models the e_0 was set to 0.1 with 4,000 warm-up steps. A batch size of 128 was used and models were trained for 20 epochs.

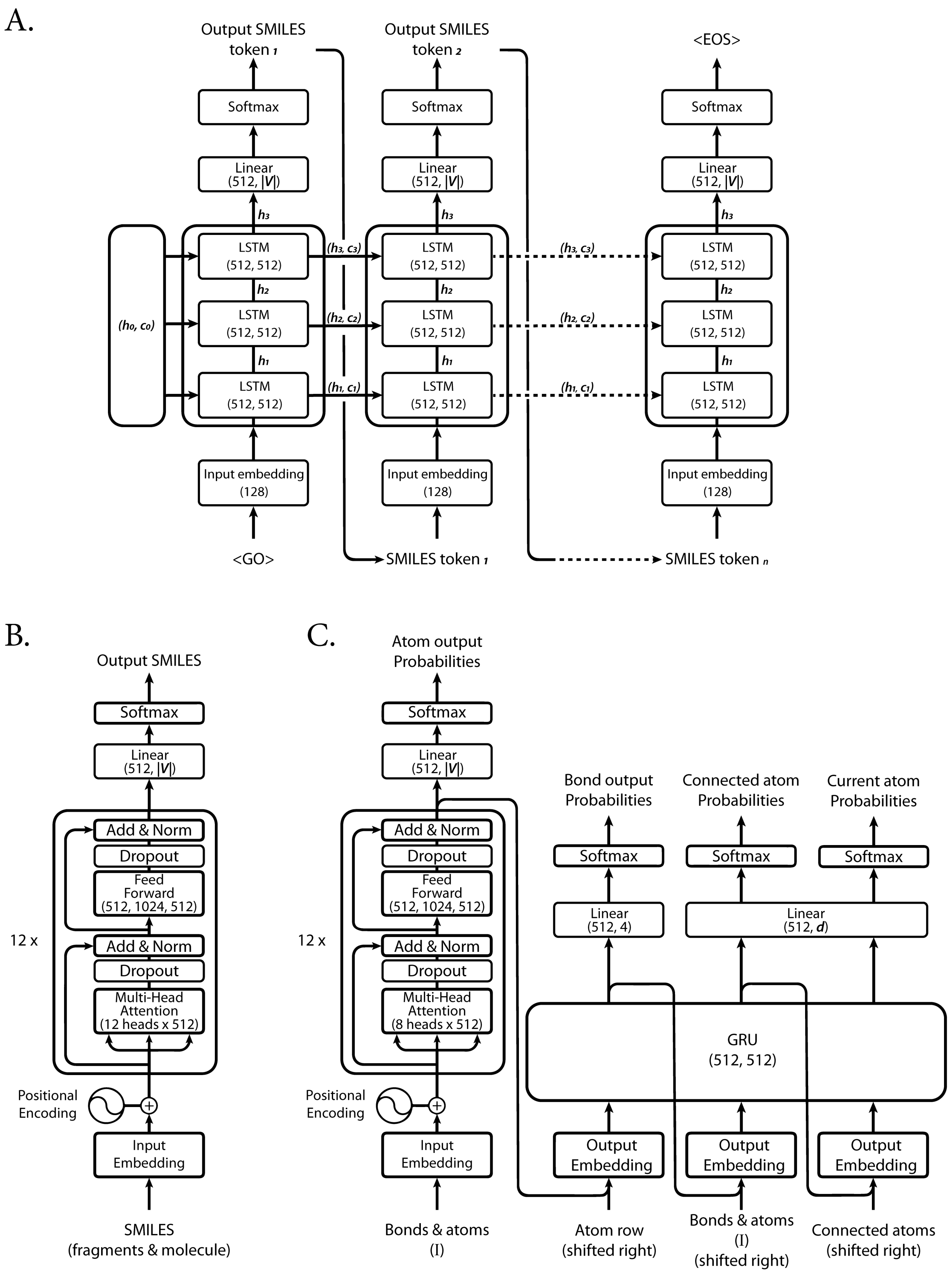

Figure S1: Illustrations of the generator architectures: sequence RNN (A), sequence transformer (B) and graph transformer (C). $|V|$ corresponds to the size of the vocabulary used. In (A), h_i and c_i correspond to the hidden state and context of the ith LSTM layer respectively. In (C), I corresponds to the index of the input word as defined in Ref. 2, and d to the number of columns in the graph matrix devoted to encoding a molecule's atoms (as illustrated by Figure 1).

RL specifications The generator parameters are updated using the policy gradient scheme, with the following objective function

$$J(\theta) = E[R^*(y_{1:T})|\theta] = \sum_{t=1}^{T} logG(y_t|y_{1:t-1}) \times R^*(y_{1:T}) \tag{S2}$$

The policy is a Gibbs distribution. By default, RL is done with a batch size of 128, for 1,000 epochs, with early stopping if there has not been an improvement for the last 50 epochs. The default exploration rate is 0.1. As described in section 2.1.3 the use of two models as exploitation network is by default only used for the RNN-based generators.

S2 Data pre-processing

Molecular standardisation first prepares molecules using the RDKit's normaliser after disconnecting metal atoms. Then the largest fragment of multi-component molecules is kept, based on the number of heavy atoms, and is uncharged. Processed molecules without carbon atoms or still containing either sodium or zinc atoms are discarded. Elemental oxygen, carbon, nitrogen and boron atoms are converted to their 'normal' counterparts, as defined by the Daylight SMILES documentation.

Molecular fragmentation is only performed when a using transformer model and relies on the BRICS[3] or RECAP[4] algorithm. Once fragments have been generated, dummy and elemental hydrogen atoms are removed. For each molecule, monoatomic fragments or substructures of others are discarded. Then the n largest fragments in terms of heavy atom count are kept. Subsequently, combinations of the kept fragments are enumerated to identify substructures of the input molecules. This last step enumerates all possible combinations of up to m of one molecule's fragments. The pairing of an input molecule with a combination of its fragments is referred to as a 'molecule-fragments pair'. By default, both n and m are equal to 4.

During fragmentation, monoatomic fragments and molecules containing a single fragment are discarded. During encoding, sequence RNN and transformer models filter out molecules with less than 10 atoms or more than 100, while graph transformers filter molecules with more than 80 bonds.

Data split is performed differently for RNN and transformers. For RNN models, pre-processed SMILES are randomly split between training and a validation set with shuffling. For the transformers, the validation set molecule-fragments pairs are sampled at random from a subset containing only a single pair per unique fragment-combination. The remaining pairs are assigned to the training set. In both cases, the number of molecules in the validation set is the minimum of either 10% of the input dataset or 10,000 entries.

RL inputs for the transformer are created either by (*i*) creating *dummy-molecule*-fragments pairs from the scaffold(s) provided by the user, or (*ii*) using a subset of preprocessed molecule-fragments pairs containing only a single pair per unique fragment-combination.

Molecular encoding For RNN models, SMILES are tokenised and each token is transformed into an integer based on an internal mapping, called vocabulary. SMILES shorter than 10 tokens or longer than 100 are filtered out. The integer sequence referred to as the 'encoded sequence', is preceded by a start token (<GO>) and ends with an end token (<EOS>). Finally, encoded sequences are padded with zeros.

For the sequence transformers, molecule-fragments pairs are encoded similarly. A custom vocabulary allows the encoding of fragment separators. Molecules and fragment combinations are encoded independently, resulting in padded and encoded outputs having 200 dimensions.

For the graph transformer, a graph matrix encoding each fragment separately and how each is linked to another is created. This graph matrix describes each atom in the molecule individually and its bonding to its neighbours as illustrated in Figure 1. Fragment indices are not defined both for atoms of the molecules not belonging to any fragment and for bonds defined in the fragment-linking section. Molecules with more than 80 bonds are discarded.

S3 Environment specifications

Table S1: List currently available scoring functions. *Available only in the API.

Property	Description
Predictive Property	A regression or classification model from QSPRpred
Ligand efficiency	$\mathrm{LE} = 1.4 \times \mathrm{pActivity}/\mathrm{N}_{\mathrm{atoms}}$
Lipophilic efficiency	$\mathrm{LipE} = \mathrm{pActivity} - \mathrm{logP}$
Tversky fingerprint similarity	Tversky similarity to reference compound calculated from fingerprints with Rdkit
Tversky graph similarity	Tversky similarity to reference compound calculated from molecular graphs
Fraggle similarity	Fraggle similarity to reference compound calculated with Rdkit
Substructure matching*	
Synthetic accessibility	Molecular complexity score calculated with Rdkit
Retrosynthetic accessibility	Binary prediction of molecule's retrosynthetic accessibility with RAscore[5]
Molecular weight	
Partition coefficient	
Quantitative estimate of drug-likeness	
Topological polar surface area	
Molar refractivity*	
Bertz molecular complexity index*	
Fraction of sp^3 carbons*	
#Hydrogen bond acceptors*	
#Hydrogen bond donors*	
#Rotable bonds*	
#Amide*	Physicochemical properties calculated with Rdkit
#Bridge*	
#Hetero atoms*	
#Heavy atoms*	
#Spiro atoms*	
#Rings*	
#Aliphatic rings*	
#Aromatic rings*	
#Saturated rings*	
#Heterocycles*	
#Valence electrons*	

Table S2: List of currently available modifier functions, their parameters and illustrations of their shapes. *These modifiers are not bounded between 0 and 1 and/or transform all objectives to maximising tasks.

**Clipped**<br>lower_x=3<br>upper_x=7<br>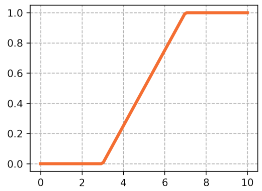	**Clipped**<br>lower_x=7<br>upper_x=3<br>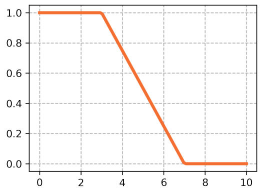	**SmoothClipped**<br>lower_x=3<br>upper_x=7<br>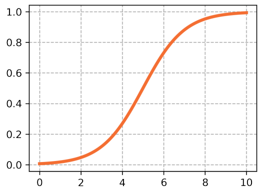	**SmoothClipped**<br>lower_x=7<br>upper_x=3<br>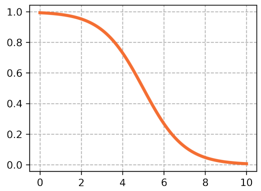
**MaxGaussian**<br>mu=5<br>sigma=1<br>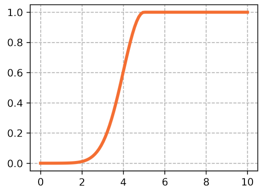	**MinGaussian**<br>mu=5<br>sigma=1<br>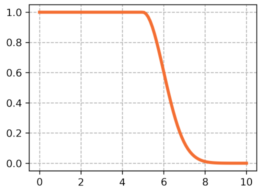	**Gaussian**<br>mu=5<br>sigma=1<br>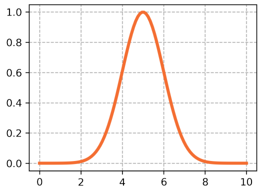	**SmoothHump**<br>lower_x=4<br>upper_x=6<br>sigma=1<br>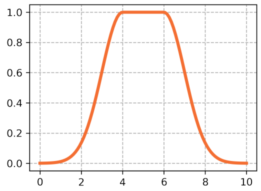
**Linear***<br>slope=1<br>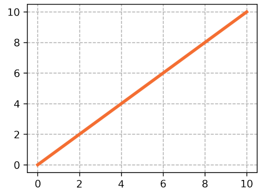	**Squared***<br>target_value=5<br>coefficient=1<br>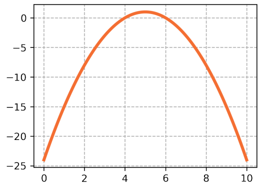	**Absolute***<br>target_value=5<br>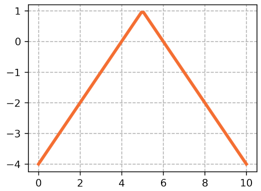	**ThresholdLinear***<br>threshold=5<br>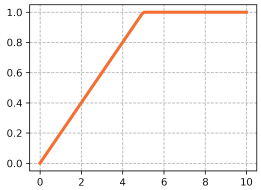

Parametric weighted sum (WS) scheme, which aggregates the scores of each objective i (R_i) for each molecule, is defined as

$$R_{\mathrm{WS}} = \sum_{i=1}^{n} \omega_i R_i \tag{S3}$$

where w_i is the weight associated to objective i and n the number of objectives. The weights for each objective are not fixed, *i.e.* hyperparameters, but dynamic, and depend on the single-objective desired ratio for each objective which is defined as

$$r_i = \frac{N_i^-}{N_i^+} \tag{S4}$$

where N_i^- and N_i^+ are the number of generated molecules with a score below and above the given threshold per objective. The final weight for each epoch is the normalized ratio defined as

$$\omega_i = \frac{r_i}{\sum_{k=1}^{n} r_k}. \tag{S5}$$

Pareto ranking-based schemes do not combine multiple objectives into one but rather search for the best trade-off between them. In Pareto ranking, one solution is only considered better than another when it is better or equal in every objective and better in at least one objective. After the dominance between all pairs of solutions is determined, the non-dominated scoring algorithm is exploited to obtain different layers of Pareto frontiers which consist of a set of solutions.[6] After assigning each molecule to a front, the compounds in each frontier are ranked based on a distance metric to increase the diversity of solutions. DrugEx proposes two distance metric formulations:

Crowding distance (PRCD) in the objective space as implemented in NSGA-II[6] with the final reward defined as

$$R_{\mathrm{CD}} = k/M \tag{S6}$$

where k is the rank of the molecule and M the total number of molecules at each iteration.

Tanimoto distance (PRTD) The molecules in the f are ranked based on their aggregated Tanimoto distance (T_d) to other molecules in the front which is defined as

$$d_f^i = f(\{T_d(m_i, m_j)\, \forall\, i \neq j \in M_f\}) \tag{S7}$$

where d_f^i is the aggregated distance measure of the compound m_i in the front f to other molecules in the same front, f is the aggregation function that can be either the minimum ($\min$), the mean (mean) or the "crowding distance" (algorithm 1) of Tanimoto distances. Then the molecules in the front are sorted from the smallest distance to the largest and the final rank is obtained by combining the ranks per front from most-*dominated* front to the most-*dominating* one:

$$k_f = \mathrm{argsort}(\{d_f^1, \ldots, d_f^{M_f}\}) \tag{S8}$$

$$k = [k_1, \ldots, k_F]. \tag{S9}$$

The final reward is defined as

$$R_{\mathrm{PR}} = \begin{cases} k/2N_{\text{undesired}} & \text{if undesired} \\ (k - N_{\text{undesired}})/2N_{\text{desired}} + 0.5 & \text{if desired} \end{cases} \tag{S10}$$

where k is the rank of the molecule, N_{desired} the number of desired molecules, *i.e.* molecules for which all objectives score above their associated thresholds, and $N_{\mathrm{undesirable}} = M - N_{\mathrm{desirable}}$. The rewards of undesired and desired solutions will be evenly distributed in]0,0.5] and]0.5,1], respectively.

Algorithm 1 Mutual similarity sort ranking

$d_f[1, ..., M_f] \leftarrow 0$
while i in M_f **do**
  $d_T \leftarrow \{T_d(m_i, m_j) \,\forall\, j \in M_f\}$
  $r_{d_T} \leftarrow \text{argsort}(d_T)$
  while j in $1, \ldots, M_f$ **do**
    $k \leftarrow r_{d_T}[j]$
    if j is 1 **then** ▷ The molecule itself gets baseline score of 0
      $d_f[k] \leftarrow d_f[k] + 0$
    else if j is N_f **then** ▷ The most dissimilar molecule gets the largest score
      $d_f[k] \leftarrow d_f[k] + 10000$
    else ▷ Others get a score based on crowding distance in the chemical space
      $d_f[k] \leftarrow d_f[k] + d_T[r_{d_T}[k+1]] - d_T[r_{d_T}[k-1]]$

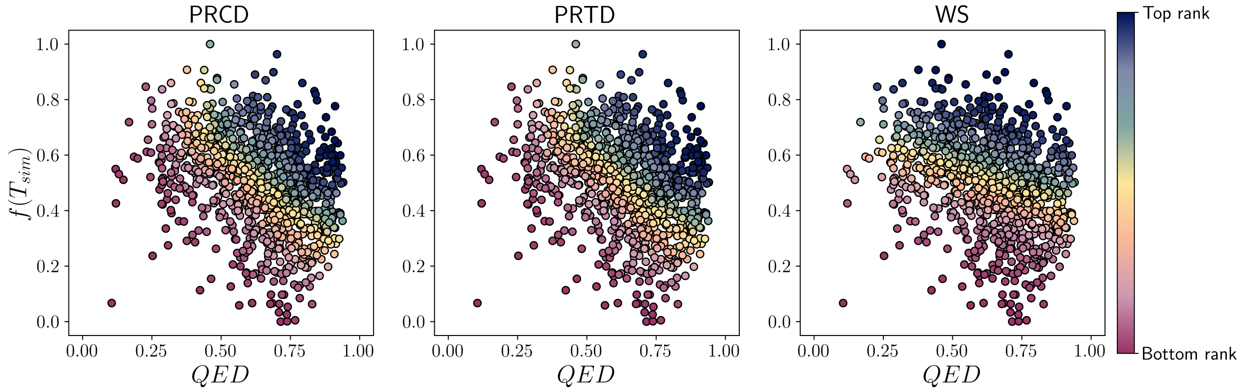

Figure S2: Illustration of two-objective ranking of 1,000 example molecules based on the maximisation of the QED and Tanimoto similarity to methotrexate. Top: Ranking with aggregation functions: Pareto-ranking with A. crowding distance (PRCD) and B. Tanimoto distance (PRTD), and C. dynamic weighted sum (WS). As the general performance is worse for the similarity than the QED, for WS the similarity has a larger weight.

S4 Sampling statistics

Table S3: Sampling performance of pretrained models.[18] Mean average values are reported. Standard deviations across independent sampling runs are reported only for the relative sampling time as all others were lower than 1.00%. Validity, accuracy, uniqueness and novelty are defined as the ratio of molecular representations that make valid molecular structures, the ratio of molecules containing the desired fragments (transformer models only), the ratio of unique molecules and the ratio of molecules not found in ZINC20[19] using the MOLBLOOM[20] Python library. The reported sampling time per molecule is relative to that of the LSTM model trained with ChEMBL version 27.[21] Sampling was independently performed on an NVIDIA A30, two separate NVIDIA GeForce RTX 2080 Ti, an NVIDIA GeForce GTX 1080 and on two separate NVIDIA GeForce RTX 3050 Ti laptop GPUs. Sample sizes were set to 100,000 molecules for RNNs and 10,000 for transformers respectively. The batch size was set to 600 for RNNs, 512 for graph transformers and 128 for SMILES transformers respectively.

Model Type	Training set	Fragmentation method	Validity	Accuracy	Uniqueness	Novelty	Relative sampling time	Ref.
SMILES GRU RNN	ChEMBL (v31)	-	1.000	-	0.996	0.999	0.705 ± 0.049	[7]
SMILES GRU RNN	Papyrus (v05.5)	-	1.000	-	0.992	0.999	0.706 ± 0.052	[8]
SMILES LSTM RNN	ChEMBL (v27)	-	0.999	-	0.600	0.865	1.000 ± 0.000	[9]
SMILES LSTM RNN	ChEMBL (v31)	-	1.000	-	0.994	0.999	0.470 ± 0.038	[10]
SMILES LSTM RNN	Papyrus (v05.5)	-	1.000	-	0.988	0.998	0.474 ± 0.050	[11]
SMILES transformer	Papyrus (v05.5)	BRICS	0.947	0.622	0.591	0.995	86.628 ± 50.843	[12]
SMILES transformer	Papyrus (v05.5)	RECAP	0.963	0.675	0.649	0.996	86.376 ± 50.629	[13]
Graph transformer	ChEMBL (v27)	BRICS	1.000	0.796	0.791	1.000	23.292 ± 10.249	[14]
Graph transformer	ChEMBL (v31)	BRICS	1.000	0.786	0.775	1.000	25.253 ± 10.373	[15]
Graph transformer	Papyrus (v05.5)	BRICS	1.000	0.762	0.751	1.000	24.694 ± 10.270	[16]
Graph transformer	Papyrus (v05.5)	RECAP	1.000	0.814	0.810	1.000	24.843 ± 10.378	[17]

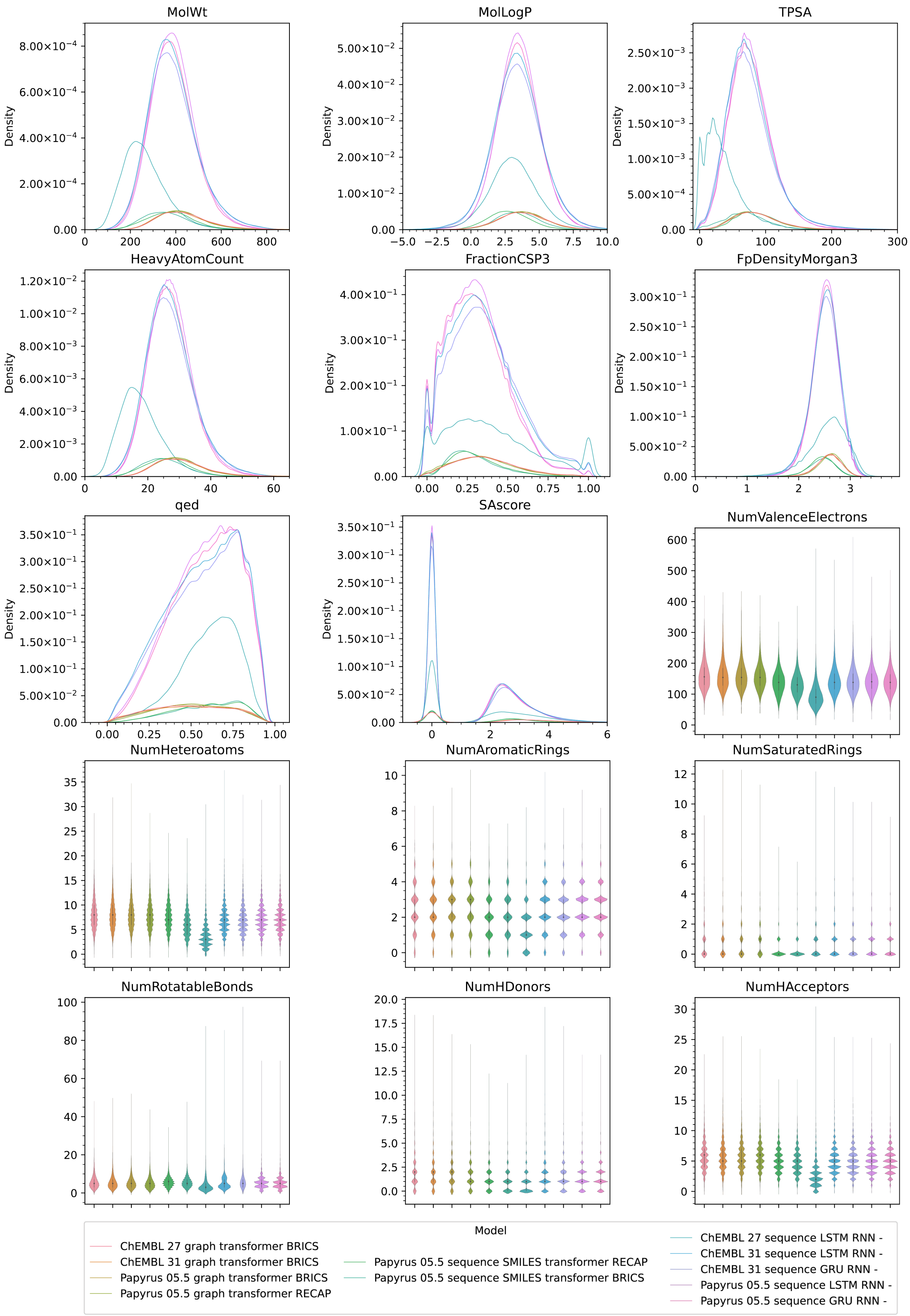

Figure S3: Distributions of molecular features of valid and unique molecules sampled in Table S3.[18] MolWt: molecular weight; MolLogP: Crippen LogP;[22] TPSA: topological polar surface area; CSP3: sp3-hybridized carbon atoms; QED: quantitative estimate of drug-likeness (higher is better);[23] SAscore: score of synthetic accessibility (higher is better).[24] All models have similar distributions of molecular features but the historical ChEMBL 27 sequence LSTM RNN which we recommend not to use.

References

(1) Radford, A.; Narasimhan, K.; Salimans, T.; Sutskever, I., et al. Improving language understanding by generative pre-training. **2018**,

(2) Liu, X.; Ye, K.; van Vlijmen, H. W. T.; IJzerman, A. P.; van Westen, G. J. P. DrugEx v3: scaffold-constrained drug design with graph transformer-based reinforcement learning. *Journal of Cheminformatics* **2023**, *15*, 24, DOI: 10.1186/s13321-023-00694-z.

(3) Degen, J.; Wegscheid-Gerlach, C.; Zaliani, A.; Rarey, M. On the Art of Compiling and Using 'Drug-Like' Chemical Fragment Spaces. *ChemMedChem* **2008**, *3*, 1503–1507, DOI: https://doi.org/10.1002/cmdc.200800178.

(4) Lewell, X. Q.; Judd, D. B.; Watson, S. P.; Hann, M. M. *Journal of Chemical Information and Computer Sciences* **1998**, *38*, 511–522, DOI: 10.1021/ci970429i.

(5) Thakkar, A.; Chadimová, V.; Bjerrum, E. J.; Engkvist, O.; Reymond, J.-L. Retrosynthetic accessibility score (RAscore) – rapid machine learned synthesizability classification from AI driven retrosynthetic planning. *Chemical Science* **2021**, *12*, 3339–3349, DOI: 10.1039/D0SC05401A.

(6) Deb, K.; Pratap, A.; Agarwal, S.; Meyarivan, T. A fast and elitist multiobjective genetic algorithm: NSGA-II. *IEEE Transactions on Evolutionary Computation* **2002**, *6*, 182–197, DOI: 10.1109/4235.996017, Conference Name: IEEE Transactions on Evolutionary Computation.

(7) Béquignon, O. J. M. DrugEx RNN-GRU pretrained model (ChEMBL31). 2023; https://doi.org/10.5281/zenodo.7550739.

(8) Béquignon, O. J. M. DrugEx RNN-GRU pretrained model (Papyrus 05.5). 2023; https://doi.org/10.5281/zenodo.7550792.

(9) Liu, X. DrugEx v2 pretrained model (ChEMBL27). 2022; https://doi.org/10.5281/zenodo.7096837.

(10) Béquignon, O. J. M. DrugEx v2 pretrained model (ChEMBL31). 2022; https://doi.org/10.5281/zenodo.7378916.

(11) Schoenmaker, L.; Béquignon, O. J. M. DrugEx v2 pretrained model (Papyrus 05.5). 2022; https://doi.org/10.5281/zenodo.7378923.

(12) Šícho, M. DrugEx pretrained model (SMILES-based; Papyrus 05.5). 2023; https://doi.org/10.5281/zenodo.7635064.

(13) Béquignon, O. J. M. DrugEx pretrained model (SMILES-based; RECAP; Papyrus 05.5). 2023; https://doi.org/10.5281/zenodo.7622774.

(14) Liu, X. DrugEx v3 pretrained model (graph-based; ChEMBL27). 2022; https://doi.org/10.5281/zenodo.7096823.

(15) Béquignon, O. J. M. DrugEx v3 pretrained model (graph-based; ChEMBL31). 2022; https://doi.org/10.5281/zenodo.7085629.

(16) Béquignon, O. J. M. DrugEx v3 pretrained model (graph-based; Papyrus 05.5). 2022; https://doi.org/10.5281/zenodo.7085421.

(17) Béquignon, O. J. M. DrugEx pretrained model (graph-based; RECAP; Papyrus 05.5). 2023; https://doi.org/10.5281/zenodo.7622738.

(18) Šícho, M.; Luukkonen, S.; van den Maagdenberg, H. W.; Schoenmaker, L.; Béquignon, O. J. M.; van Westen, G. J. P. Sampling timings of DrugEx pretrained models. 2023; https://doi.org/10.5281/zenodo.7928362.

(19) Irwin, J. J.; Tang, K. G.; Young, J.; Dandarchuluun, C.; Wong, B. R.; Khurelbaatar, M.; Moroz, Y. S.; Mayfield, J.; Sayle, R. A. ZINC20—A Free Ultralarge-Scale Chemical Database

for Ligand Discovery. *Journal of Chemical Information and Modeling* **2020**, *60*, 6065–6073, DOI: 10.1021/acs.jcim.0c00675.

(20) White, A. D. molbloom: quick assessment of compound purchasability with bloom filters. 2022; https://github.com/whitead/molbloom.

(21) Liu, X.; Ye, K.; van Vlijmen, H. W. T.; Emmerich, M. T. M.; IJzerman, A. P.; van Westen, G. J. P. DrugEx v2: de novo design of drug molecules by Pareto-based multi-objective reinforcement learning in polypharmacology. *Journal of Cheminformatics* **2021**, *13*, 85, DOI: 10.1186/s13321-021-00561-9.

(22) Wildman, S. A.; Crippen, G. M. Prediction of Physicochemical Parameters by Atomic Contributions. *Journal of Chemical Information and Computer Sciences* **1999**, *39*, 868–873, DOI: 10.1021/ci990307l.

(23) Wildman, S. A.; Crippen, G. M. Prediction of Physicochemical Parameters by Atomic Contributions. *Journal of Chemical Information and Computer Sciences* **1999**, *39*, 868–873, DOI: 10.1038/nchem.1243.

(24) Bickerton, G. R.; Paolini, G. V.; Besnard, J.; Muresan, S.; Hopkins, A. L. Quantifying the chemical beauty of drugs. *Journal of Cheminformatics* **2012**, *4*, 90–98, DOI: 10.1186/1758-2946-1-8.
